# Wild type transthyretin cardiac amyloidosis in a young individual: A case report

**DOI:** 10.1097/MD.0000000000025462

**Published:** 2021-04-30

**Authors:** Shreya Ghosh, Dibbendhu Khanra, Vinay Krishna, Ashwani Kumar Thakur

**Affiliations:** a Department of Biological Sciences and Bioengineering, Indian Institute of Technology Kanpur, Uttar Pradesh; b Department of Cardiology, All India Institute of Medical Sciences (AIIMS) Rishikesh, Uttarakhand; c Department of cardiac surgery, Laxmipat Singhania Institute of Cardiology & Cardiac Surgery, Ganesh Shankar Vidyarthi Memorial Medical College Kanpur, Uttar Pradesh, India.

**Keywords:** cardiac amyloidosis, case report, transthyretin, wildtype, young

## Abstract

**Rationale:**

Senile systemic amyloidosis, a disease of elderly is caused by amyloid deposition of wild-type transthyretin. The symptoms often overlap with other heart diseases. Hence it is either misdiagnosed or considered as a normal aging process in majority of cases.

**Patient concerns:**

We present a young patient of wild-type transthyretin amyloidosis, contradicting its only senile presence. The 34-year-old man presented with dyspnoea on exertion. He was suffering from hypertension for consecutive 3 years.

**Diagnosis:**

Echocardiography demonstrated left ventricular hypertrophy with reduced global longitudinal strain and apical sparing. Congo red staining and immuno-histochemical staining of the abdominal fat biopsy confirmed transthyretin amyloid deposition. Genetic analysis revealed absence of any mutant variant/s of transthyretin gene, confirming wild-type transthyretin amyloidosis.

**Intervention:**

A combination of amlodipine 5 mg, telmisartan 40 mg, and chlorthalidone 12.5 mg once daily was given to control the blood pressure of the patient.

**Outcome:**

Blood pressure was controlled but he continued to have exertional dyspnoea. The patient expired in December 2019.

**Lessons:**

A systematic diagnosis for wild type transthyretin amyloid cardiomyopathy (ATTR-CM) shall be considered in young cardiac patients suffering from cardiac distress with unknown etiology.

## 1 Introduction

Cardiac amyloidosis is characterized by the extracellular deposition of some amyloid precursor proteins in the myocardium of heart. The systemic form of cardiac amyloidosis is mainly driven by either the misfolded monoclonal immunoglobulin light chains (kappa and lambda) or transthyretin.^[1]^ The symptoms of cardiac amyloidosis often overlap with symptoms of other cardiovascular diseases mostly hypertrophic cardiomyopathy. Hence, in majority of cases, it is often overlooked and remains undiagnosed. In the recent years, incidence of transthyretin related amyloidosis has increased across the world.^[2]^ However, there are no published reports on transthyretin-related cardiac amyloidosis from India. In this direction, researchers in collaboration with the clinicians have started sensitising for the need of systematic diagnosis of amyloidosis in India.^[3]^ With this effort, for the first time, we are reporting a case of senile systemic amyloidosis in a young individual from India.

## 2 Case report

**Figure 1:**
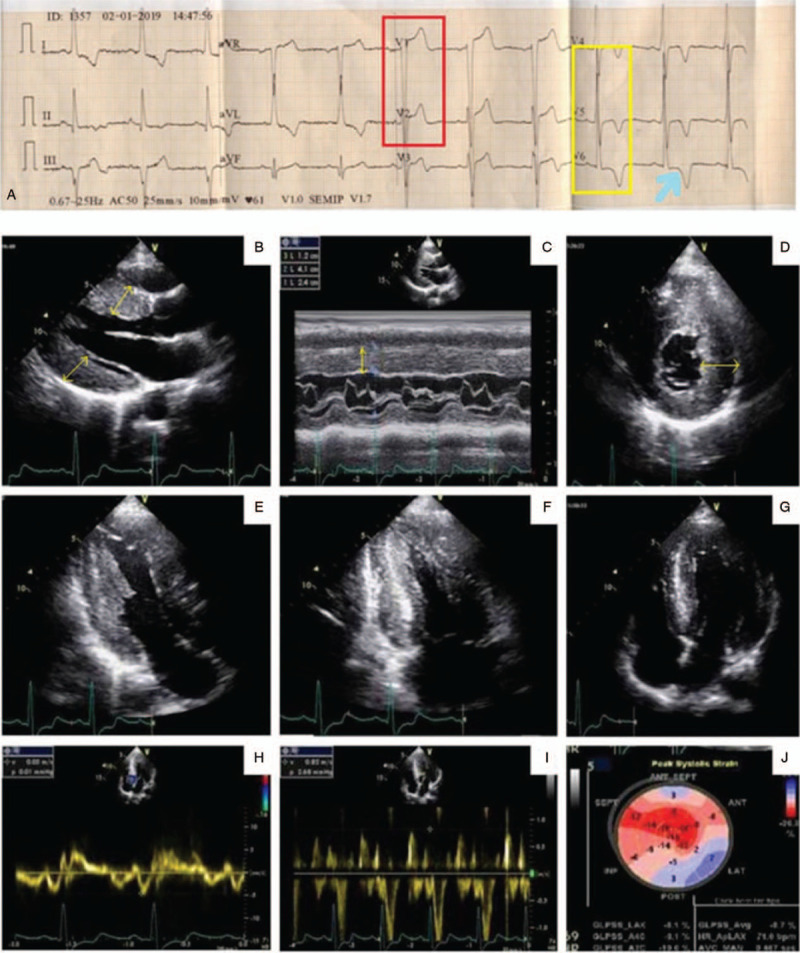
Surface electrocardiogram of the patient showing evidence of left ventricular hypertrophy (red box and yellow box) with strain pattern (arrow) (A). Trans-thoracic echocardiography showing evidence of asymmetric LVH (B) with inter-ventricular septal diameter of 2.4 cm (yellow arrow) in diastole (C) along with speckled pattern in the myocardium (D, E) without any evidence of left ventricular outflow tract gradient or systolic anterior motion of anterior mitral valve leaflet (F, G). Tissue Doppler study revealed grade 2 left ventricular diastolic dysfunction (H, I). Strain echocardiography using speckled tracking revealed marked diminution of global longitudinal strain with apical sparing (J). LVH = left ventricular hypertrophy.

**Figure 2:**
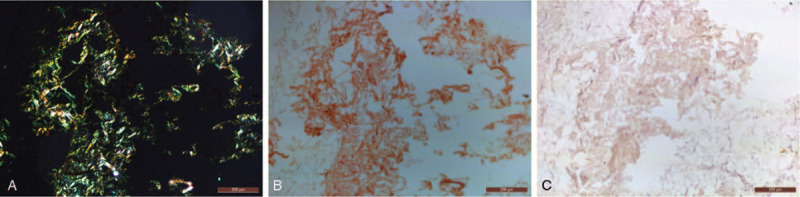
Microscopy images of Congo red stained abdominal fat biopsy tissue specimen under polarized light (A) and unpolarized light (B) confirming amyloidosis. Light microscopy image of anti-TTR antibody stained fat tissue specimen showing positive reaction in amyloid enriched areas (C). Scale bar: 200 μm.

A 34-year-old male patient from North India presented with exertional dyspnea stage II (New York Heart Association) without any chest pain or pedal swelling. He was hypertensive for 3 years, not controlled with amlodipine 5 mg once daily. There was no family history of inflammatory disease, heart failure or premature cardiac death. His pulse rate was 84 beats per minute with all peripheral pulses palpable. Jugular venous pressure was not elevated. There was no lymphadenopathy and organomegaly, suggested by abdominal ultrasonography. Routine hematological and biochemical parameters including renal and liver function test (LFT) profile were within the normal range. The electrocardiogram of the patient suggested left ventricular hypertrophy (LVH) with strain pattern and sinus rhythm (Fig. 1A). Trans-thoracic echocardiography showed evidence of asymmetric LVH (Fig. 1B) with interventricular septal diameter of 2.4 cm in diastole (Fig. 1C) and sparkling pattern of the myocardium (Fig. 1D, E). However there was no significant left ventricular outflow tract gradient or systolic anterior motion of anterior mitral valve leaflet (Fig. 1F, G). Tissue doppler study revealed grade 2 left ventricular diastolic dysfunction with *E*/*e*′ ratio of 20 (Fig. 1H, I). Strain echocardiography using speckled tracking showed a reduced global longitudinal strain of –8.7% with apical sparing (Fig. 1J), classically known as “cherry on top” pattern, suggesting cardiac amyloidosis. Ejection fraction to strain ratio (EFSR) was calculated as 5.51. Left atrial volume index was 40 mL/m^2^ with an evidence of mild mitral regurgitation and trivial tricuspid regurgitation, suggestive of diastolic dysfunction. Coronary angiogram showed normal epicardial coronary arteries. Nerve conduction velocity study revealed no evidence of carpal tunnel syndrome. Cardiac magnetic resonance imaging (MRI) was performed but it showed no late gadolinium enhancement (LGE). Immunofixation electrophoresis showed no M spike, but a polyclonal increase in immunoglobulin G was found (Supplemental Digital Content [Fig. S1]). Serum free light chain assay showed an increased immunoglobulin *κ* light chain and *κ*/*λ* ratio (Supplemental Digital Content [Table S1]). Bone marrow examination was not done because the patient did not give consent for bone marrow biopsy. Histopathological examination of the abdominal fat biopsy specimen showed the presence of extensive amyloid deposits, exhibiting characteristic apple-green birefringence under polarized light (Fig. 2A) upon Congo red staining (Fig. 2B). Immuno-histochemical staining of fat biopsy specimen with the anti-transthyretin antibody (Catalog no. sc-377517), raised against the amino acid stretch 91–129 of human transthyretin protein, showed positive results (Fig. 2C). But no positive reaction was observed upon staining with antibody against immunoglobulin light chain κ (Catalog no. PA5-16647) (Supplemental Digital Content [Fig. S4]). However, genetic analysis revealed absence of any mutation in the transthyretin gene (Supplemental Digital Content [Table S2, S3] and [Fig. S2, S3]). The patient's blood pressure was controlled with amlodipine 5 mg, telmisartan 40 mg, and chlorthalidone 12.5 mg once daily without any symptomatic hypotension. However, the patient continued to have exertional dyspnoea. Unfortunately, he died in December, 2019.

## 3 Discussion

Senile systemic amyloidosis, caused by wildtype transthyretin (ATTRwt) is considered as the disease of aged individuals with more severity in octagenerians.^[4]^ However, the patient being young makes this case more fascinating than the earlier published reports where the youngest case reported was 47 years old.^[5]^ Reduced global longitudinal strain (>–15.1%) with apical sparing pattern and an EFSR value of >4.1 are reported echocardiogram features for cardiac amyloidosis. These features differentiate it from other causes of LVH by 72%, 82%, and 92% specificity respectively.^[6,7]^ Echocardiogram of our patient also reflected these same features. The presence of LVH in echocardiogram and pseudo-infarct pattern along with sinus rhythm in electrocardiogram are common features found in transthyretin amyloid cardiomyopathy (ATTR-CM) patients.^[8,9]^ Our patient also showed evidences of asymmetric LVH in the echocardiogram and pseudo-infarct pattern along with sinus rhythm in the electrocardiogram, suggesting ATTR-CM. Moreover, presence of “cherry on top” pattern in the bull eye plot in the 2 dimensional speckle tracking echocardiographic findings of our patient also provided significant cues for ATTR-CM.^[2]^ Increased interventricular septal wall thickness is often considered as a strong indicator for ATTR amyloidosis despite of the absence of low voltage in the electrocardiogram.^[10]^ This was consistent with the echocardiographic findings of our patient too. LGE pattern might be absent in some ATTRwt cases and was also true for our case.^[11]^ The presence of monoclonal gammopathy along with renal impairment and macroglossia is highly implicated in case of immunoglobulin light chain (AL) amyloidosis.^[4]^ However, our patient did not show such features. Besides, abnormal serum FLC *κ*/*λ* ratio with an elevated FLC level in the absence of a monoclonal protein is often found in ATTRwt patients.^[12,13]^ Our patient also showed an elevated kappa light chains and *κ*/*λ* ratio without M spike, suggesting involvement of wildtype transthyretin. Endomyocardial biopsy is the gold standard for confirming the presence of amyloid deposits in heart by classical Congo red dye staining. But, it involves certain risks as well as expertise in handling.^[14]^ However, literature suggests that abdominal fat biopsy can also type ATTR amyloidosis with significant confidence.^[15,16]^ In case of our patient, histopathological examination of the abdominal fat biopsy specimen revealed extensive amyloid deposits derived from transthyretin. It was confirmed as wild type by genetic analysis.

## 4 Conclusion

To the best of our knowledge, this is the first report of transthyretin-related amyloidosis from India. So far, this is also the youngest case of wild-type transthyretin reported in the world. This study will stimulate the cardiologists to carry a systematic approach to diagnose wild-type transthyretin-related amyloidosis not only in elderly but in young patients as well.

## Acknowledgments

AKT dedicate this article with gratitude to the patient who gave consent to investigate him for research. SG thanks MHRD funding for her PhD fellowship. The authors gratefully acknowledge Prof Saravanan Matheshwaran for allowing the usage of PCR machine. They sincerely thank Dr Avinash Yashwant Gahane from IIT Kanpur, Dr Saurabh Agarwal and Dr Chayanika Kala from LPS Institute of Cardiology, Kanpur for their kind support for biopsy and histology consultation.

## Author contributions

**Conceptualization:** Ashwani Kumar Thakur.

**Data curation:** Shreya Ghosh, Dibbendhu Khanra.

**Formal analysis:** Shreya Ghosh, Dibbendhu Khanra, Ashwani Kumar Thakur.

**Project administration:** Ashwani Kumar Thakur.

**Resources:** Vinay Krishna, Ashwani Kumar Thakur.

**Supervision:** Vinay Krishna, Ashwani Kumar Thakur.

**Visualization:** Shreya Ghosh, Dibbendhu Khanra.

**Writing – original draft:** Shreya Ghosh, Dibbendhu Khanra, Ashwani Kumar Thakur.

**Writing – review & editing:** Shreya Ghosh, Dibbendhu Khanra, Vinay Krishna, Ashwani Kumar Thakur.

## Supplementary Material

Supplemental Digital Content

## Supplementary Material

Supplemental Digital Content

## Supplementary Material

Supplemental Digital Content

## Supplementary Material

Supplemental Digital Content

## Supplementary Material

Supplemental Digital Content

## Supplementary Material

Supplemental Digital Content

## Supplementary Material

Supplemental Digital Content
