# Supplementary figures and images for: Wild type transthyretin cardiac amyloidosis in a young individual: A case report
Source: Medicine (Baltimore). 2021 Apr 30;100(17):e25462. doi: 10.1097/MD.0000000000025462 (PMC8084012; doi:10.1097/MD.0000000000025462)

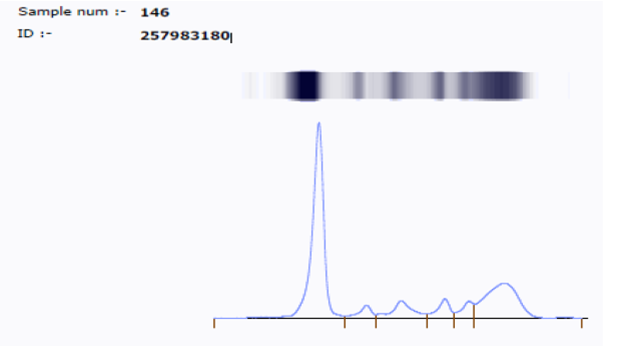

Supplement: Supplemental Digital Content [file medi-100-e25462-s001.tiff]

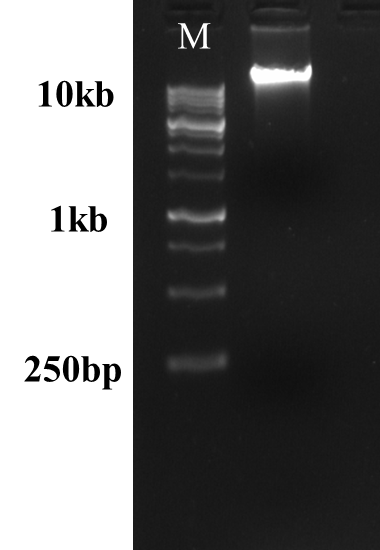

Supplement: Supplemental Digital Content [file medi-100-e25462-s006.tiff]

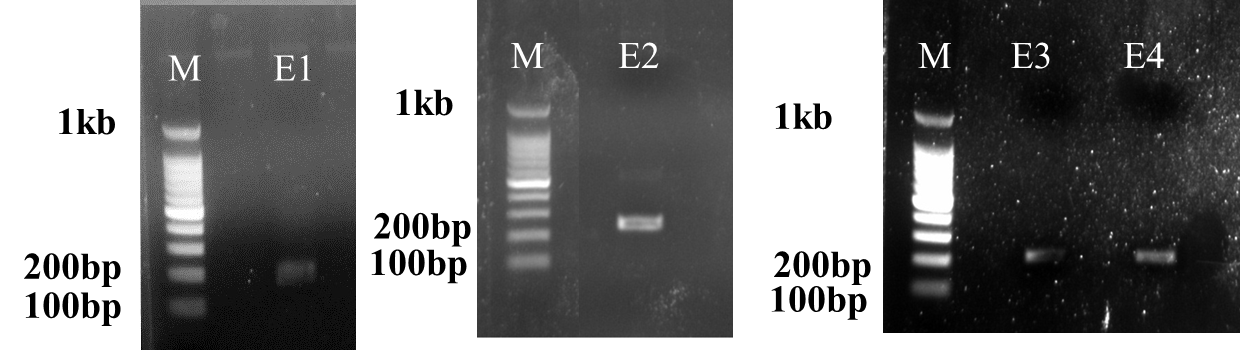

Supplement: Supplemental Digital Content [file medi-100-e25462-s007.tiff]
