# Supplementary material for: Wild type transthyretin cardiac amyloidosis in a young individual: A case report
Source: Medicine (Baltimore). 2021 Apr 30;100(17):e25462. doi: 10.1097/MD.0000000000025462 (PMC8084012; doi:10.1097/MD.0000000000025462)
Supplement: Supplemental Digital Content [file medi-100-e25462-s002.doc]

**Table S1** List of Multiple Myeloma Screening Panel (in the absence of M spike, a κ: λ ratio up to 2.5 can typically be considered normal (Witteles RM *et.al*. JACC: Heart Failure 2019)).

| **Parameters** | **Observed values** | **Biological reference interval** |
| --- | --- | --- |
| **Protein Electrophoresis Serum (SPE)** |  |  |
| 1.Protein total | 9.20 g/dl | 6.40-8.30 g/dl |
| 2.Albumin | 4.81 g/dl | 3.60-5.50 g/dl |
| 3. Alpha 1 globulin | 0.34 g/dl | 0.20-0.40 g/dl |
| 4.Alpha 2 globulin | 0.74 g/dl | 0.5-1.0 g/dl |
| 5.Beta 1 globulin | 0.50 g/dl | 0.50-1.10g/dl |
| 6.Beta 2 globulin | 0.41g/dl | 0.30-0.60 g/dl |
| 7.Gamma globulin | **2.40 g/dl** | 0.7-1.60 g/dl |
| 8.A:G Ratio | 1.10 | 0.9-2.0 |
| 9.M spike | Not seen | - |
| **Immunoglobulin profile** |  |  |
| 1.Immunoglobulin IgG, serum | **2399.0 mg/dl** | 700-1600 mg/dl |
| 2.Immunoglobulin IgM, serum | 45 mg/dl | 40-230 mg/dl |
| 3.Immunoglobulin IgA, serum | 198 mg/dl | 70-400 mg/dl |
| **Free light chain assay, serum** |  |  |
| 1.Kappa free light chain | **43**.**90 mg/L** | 3.30-19.40 mg/L |
| 2. Lambda free light chain | 19.50 mg/L | 5.71- 26.30 mg/L |
| 3.Kappa/lambda ratio | **2.251** | 0.26-1.65 |
| **BENCE JONES Protein, urine, Qualitative** | Absent | - |
