# Supplementary material for: Wild type transthyretin cardiac amyloidosis in a young individual: A case report
Source: Medicine (Baltimore). 2021 Apr 30;100(17):e25462. doi: 10.1097/MD.0000000000025462 (PMC8084012; doi:10.1097/MD.0000000000025462)

**Figure S4** Light microscopy image of anti-immunoglobulin κ antibody stained fat tissue specimen showing negative reactivity (A) in amyloid enriched Congo red positive areas (B). Scale bar**:** 200 µm


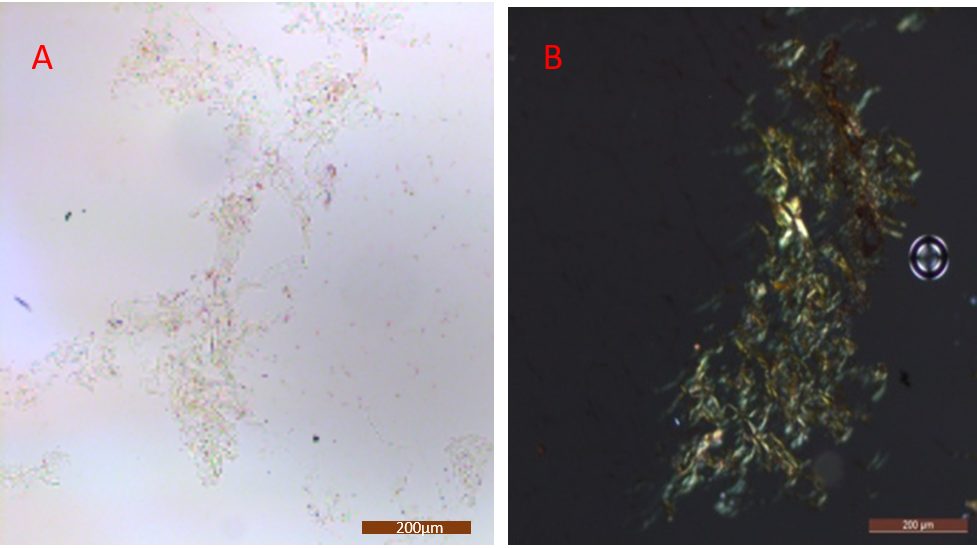

Supplement: Supplemental Digital Content [file medi-100-e25462-s003.doc]
