# Supplementary material for: Wild type transthyretin cardiac amyloidosis in a young individual: A case report
Source: Medicine (Baltimore). 2021 Apr 30;100(17):e25462. doi: 10.1097/MD.0000000000025462 (PMC8084012; doi:10.1097/MD.0000000000025462)
Supplement: Supplemental Digital Content [file medi-100-e25462-s004.doc]

**Table S2** Detail of the primers designed for amplifying four exons of transthyretin gene (transthyretin gene sequence (sequence of four exons) was adapted from NCBI, Gene Id: 7276. Both forward and reverse primers for respective four exons were designed, using the adapted sequence for respective four exons as the template).

| **Exon No** | **Sequence (5’ to 3’)** | **Length** | **GC%** | **Product Length** |
| --- | --- | --- | --- | --- |
| Exon 1 Forward | AGCAGCCTAGCTCAGGAGAA | 20 | 55 | 178 |
| Exon 1 Reverse | AGGAATGGGATGTCACAGAAAC | 22 | 45 | 178 |
| Exon 2 Forward | CTCACGTGTCTTCTCTACACCC | 22 | 55 | 224 |
| Exon 2 Reverse | TCTCTACCAAGTGAGGGGCA | 20 | 55 | 224 |
| Exon 3 Forward | GCCATGCCATTTGTTTCCTC | 20 | 50 | 216 |
| Exon 3 Reverse | ACCCTCGAAGGTCTGTATACTC | 22 | 50 | 216 |
| Exon 4 Forward | CTGTCACGTTTTTCGGGCTCT | 21 | 52 | 206 |
