# Supplementary material for: Wild type transthyretin cardiac amyloidosis in a young individual: A case report
Source: Medicine (Baltimore). 2021 Apr 30;100(17):e25462. doi: 10.1097/MD.0000000000025462 (PMC8084012; doi:10.1097/MD.0000000000025462)
Supplement: Supplemental Digital Content [file medi-100-e25462-s005.doc]

**Table S3** Trimmed sequences of the four amplified exons of transthyretin gene obtained from patient’s genomic DNA.

| Exon 1 forward | CACAGAAGTCCACTCATTCTTGGCAGGATGGCTTCTCATCGTCTGCTCCTCCTCTGCCTTGCTGGACTGGTATTTGTGTCTGAGGCTGGCCCTACGGTGAGTGTTTCTGTGACATCCCATTCC |
| --- | --- |
| Exon 1 reverse | TCCAGCAAGGCAGAGGAGGAGCAGACGATGAGAAGCCATCCTGCCAAGAATGAGTGGACTTCTGTGATGGCTGCTCCCAGCCTGGGGCTTTTATACTCACTTCTCCTGAGCTAGG |
| Exon 2 forward | GATGGTCAAGTTCTAGATGCTGTCCGAGGCAGTCCTGCCATCAATGTGGCCGTGCATGTGTTCAGAAAGGCTGCTGATGACACCTGGGAGCCATTTGCCTCTGGGTAAGTTGCCAAAGAACCCTCCCACAGGACTTGGTTTTATCTTCCCGTTTGCCCCTCACTTG |
| Exon 2 reverse | GGGTTCTTTGGCACTTACCCAGAGGCAAATGGCTCCCAGGTGTCATCAGCAGCCTTTCTGAACACATGCACGGCCACATTGATGGCAGGACTGCCTCGGACAGCATCTAGAACTTTGACCATCAGAGGACACTTGGATTCACCGGTGCCCTGGGTGTAGA |
| Exon 3 forward | TAGGAAACCAGTGAGTCTGGAGAGCTGCATGGGCTCACAACTGAGGAGGAATTTGTAGAAGGGATATACAAAGTGGAAATAGACACCAAATCTTACTGGAAGGCACTTGGCATCTCCCCATTCCATGAGCATGCAGAGGTGAGTATACAGACCTTCGAGGGTAA |
| Exon 3 reverse | GCCTTCCAGTAAGATTTGGTGTCTATTTCCACTTTGTATATCCCTTCTACAAATTCCTCCTCAGTTGTGAGCCCATGCAGCTCTCCAGACTCACTGGTTTTCCTATAAGGTGTGAAAGTCTGGATTAAGTTACGCATGGAGGAAACAGATG |
| Exon 4 forward | TTCACAGCCAACGACTCCGGCCCCCGCCGCTACACCATTGCCGCCCTGCTGAGCCCCTACTCCTATTCCACCACGGCTGTCGTCACCAATCCCAAGGAATGAGGGACTTCTCCTCCAGTGGACCTGAAGGACGAGGG |
| Exon 4 reverse | GGTGACGACAGCCGTGGTGGAATAGGAGTAGGGGCTCAGCAGGGCGGCAATGGTGTAGCGGCGGGGGCCGGAGTCGTTGGCTGTGAATACCACCTATGAGAGAAGACAGACAGATCCATTTCCACCAGAGCCCGAAAA |
